# Emergency department impaired adherence to personal protective equipment donning and doffing protocols during the COVID-19 pandemic

**DOI:** 10.1186/s13584-021-00477-7

**Published:** 2021-07-19

**Authors:** Tomer Lamhoot, Noa Ben Shoshan, Hagit Eisenberg, Gilad Fainberg, Mansour Mhiliya, Neta Cohen, Orly Bisker-Kassif, Orly Barak, Carolyn Weiniger, Tali Capua

**Affiliations:** 1 Medical Technology and Simulation Center, Tel Aviv Sourasky Medical Center, affiliated to Ministry of Health, Tel Aviv, Israel; 2 Pediatric Emergency Medicine, The hospital for Sick Children, Toronto, Canada; 3 Pediatric Emergency Medicine, Dana-Dwek Children’s Hospital, Tel Aviv, Israel, affiliated to the Sackler Faculty of Medicine, Tel Aviv University, Tel Aviv, Israel; 4 Department of Geriatrics, Tel Aviv Sourasky Medical Center, Tel Aviv, Israel affiliated to the Sackler Faculty of Medicine, Tel Aviv University, Tel Aviv, Israel; 5 Department of Anesthesia, Tel Aviv Sourasky Medical Center, Tel Aviv, Israel, affiliated to the Sackler Faculty of Medicine, Tel Aviv University, Tel Aviv, Israel

**Keywords:** Personal protective equipment, COVID-19, Emergency department

## Abstract

**Objectives:**

Coronavirus Disease 2019 (COVID-19) is a highly infectious viral pandemic that has claimed the lives of millions. Personal protective equipment (PPE) may reduce the risk of transmission for health care workers (HCWs), especially in the emergency setting. This study aimed to compare the adherence to PPE donning and doffing protocols in the Emergency Department (ED) vs designated COVID-19 wards and score adherence according to the steps in our protocol.

**Design:**

Prior to managing COVID-19 patients, mandatory PPE training was undertaken for all HCWs. HCWs were observed donning or doffing COVID-19 restricted areas.

**Setting:**

Donning and doffing was observed in COVID-19 designated Emergency department and compared to COVID-19 positive wards.

**Participants:**

All HCWs working in the aforementioned wards during the time of observation.

**Results:**

We observed 107 donning and doffing procedures (30 were observed in the ED). 50% HCWs observed donned PPE correctly and 37% doffed correctly. The ED had a significantly lower mean donning score (ED: 78%, Internal: 95% ICU: 96%, *p* < 0.001); and a significantly lower mean doffing score (ED: 72%, Internal: 85% ICU: 91%, *p* = 0.02).

**Conclusions:**

As hypothesized, HCWs assigned to the designated ED wing made more protocol deviations compared with HCWs positive COVID-19 wards. Time management, acuity, lack of personnel, stress and known COVID-19 status may explain the lesser adherence to donning and doffing protocols. Further studies to assess the correlation between protocol deviations in use of PPE and morbidity as well as improvement implementations are required. Resources should be invested to ensure PPE is properly used.

**Supplementary Information:**

The online version contains supplementary material available at 10.1186/s13584-021-00477-7.

## Introduction

Personal protective equipment (PPE) is defined as barrier clothing, gloves, eye protection and/or headgear designed to protect from a potential hazardous exposure. In medicine, these exposures are typically either infectious diseases or toxins (1). The transmission of the Severe acute respiratory syndrome coronavirus 2 *(*SARS*-*CoV*-*2*)*, and its disease, COVID-19, is presumed to be primarily through droplets and fomites, although viral particles have also been found in feces of seropositive patients. A recent study by Van Doremalen et al. suggested aerosol and fomite as a mode of SARS-CoV-2 transmission, and reinforced the reported high spread rate (2). In hospital settings, performing aerosol-generating procedures (e.g. intubation, suction, bronchoscopy and cardiopulmonary resuscitation) facilitated patient-to-healthcare worker (HCW) transmission (3, 4).

The COVID-19 pandemic has highlighted the need for appropriate PPE to reduce transmission risks for HCWs caring for COVID-19 patients (5). Reports from Italy’s experience dealing with COVID-19 suggest that up to 20% of healthcare professionals dealing with COVID-19 patients became infected with the virus, with some reported deaths (6). China’s experience also found 41% of patients in one hospital acquired COVID-19 in hospital (7). Furthermore, Adams et al. reported that 3000 HCWs were infected with COVID-19 and 22 died (8).

International guidelines exist for HCWs regarding PPE use to minimize COVID-19 transmission (9, 10), and precautions during donning and doffing are recommended in all guidelines (4).

PPE donning and doffing entails specific steps, reportedly taking 7 to 15 min for donning and 14 to 23 for doffing (11).

A review of the literature between 2014 to 2020 reported HCW protocol deviations both during donning and doffing when using PPE, with consequential contamination risks (5, 12–15).

To the best of our knowledge, in-situ PPE donning and doffing has not been studied in designated COVID-19 wards. Our objective was to assess adherence of PPE donning and doffing protocols, and compare Emergency Department (ED) assessments to non-emergency departments. Due to the suspected status of the patients, high volume of patients with variable degrees of acuity and inappropriate staff to patient ratios, we hypothesized that protocol deviations would be more likely to occur in COVID-19 ED wing and less likely in COVID-19 confirmed floors (i.e. Intensive Care Unit - ICU and internal medicine ward).

## Methods

### Study design

This study comprised three groups; teams entering and exiting the COVID-19 suspected ED wing, COVID-19 confirmed ICU and COVID-19 internal medicine ward.

### Study protocol

A checklist was created, based on the hospital protocol for donning and doffing PPE (Appendices 1a and 1b). This protocol is based on the guidelines of the World Health Organization (WHO) and the Centers for Disease Control and Prevention (CDC) regarding PPE for COVID-19 (9, 10). Donning was assessed in eleven steps, and doffing in eight steps with an extra step for those wearing eyeglasses. Mandatory PPE donning and doffing training was undertaken for all HCWs between March 4 and April 10, 2020. Prior to managing COVID-19 suspected/positive patients, all HCWs participated in a group, frontal demonstration and practice. A short video clip was distributed to all hospital staff. A poster (Additional file 1) was placed in all departments, and the poster and video were available via the hospital COVID-19 mobile app.

Observers were trained by the epidemiology department to teach and observe donning and doffing. Observations were conducted between April 20th and April 26th, 2020. Time slots for observations were chosen randomly but included all hours of the day, all days of the week. The HCWs were not alerted to observations taking place to reduce possible procedural bias however, all wards had PPE adherence staff in place during the time of the observations.

A sample donning and doffing event was calculated by all members of the team to assess intra-team validity. Donning and doffing was scored according to the correct performance of each item thus the maximum score for donning was 11 and for doffing was 8 (9 if wearing eyeglasses). We used a detailed checklist to minimize intra-observer variation.

Our Institutional Review Board waived the need for approval and consent due to the nature of the project.

### Study setting, population and sample size

This study was performed in a-1100 bed, tertiary care center between March 4 and April 26, 2020 as part of a quality improvement project during the COVID-19 pandemic.

Observations of 107 HCWs were performed in COVID-19 designated departments - internal medicine ward (Internal; 62 HCWs) and intensive care unit (ICU) for confirmed positive COVID-19 cases (15 HCWs) and a designated emergency department (ED) wing for suspected or confirmed COVID-19 cases (30 HCWs). All three departments had designated areas for both donning and doffing. Observations were recorded according to the checklist (Appendices 1a and 1b).

Inclusion criteria: HCWs assigned to COVID-19 designated wards and ED, entering or exiting the restricted area when observers were present.

### Data analysis

All checklist records were collected via Microsoft Excel 2016 (version 16.0.4266.1001). Mean donning and doffing score were our primary outcome. Generalized Linear Models (GLM) were calculated and analyzed with R software (version 3.6.1) to predict PPE donning and doffing mean score based on department type, worker type and their interaction.

## Results

One hundred and seven HCW observations were made; 56 during donning and 51 during doffing out of which, 19 and 11 HCWs in the ED respectively. HCWs included: nurses (*n* = 50), attending physicians (*n* = 4), resident doctors (*n* = 11), intern doctors (*n* = 7), physiotherapists (*n* = 3), janitors (n = 4), patient transport staff (*n* = 16), X-Ray technicians (n = 7), paramedics (n = 1), and administrative staff (*n* = 2), in three different departments: COVID-19 positive internal medicine department (Internal; *n* = 62), suspected COVID-19 designated Emergency Department (ED; *n* = 30), and the COVID-19 positive Intensive care unit (ICU; *n* = 15).

**Fig. 1:**
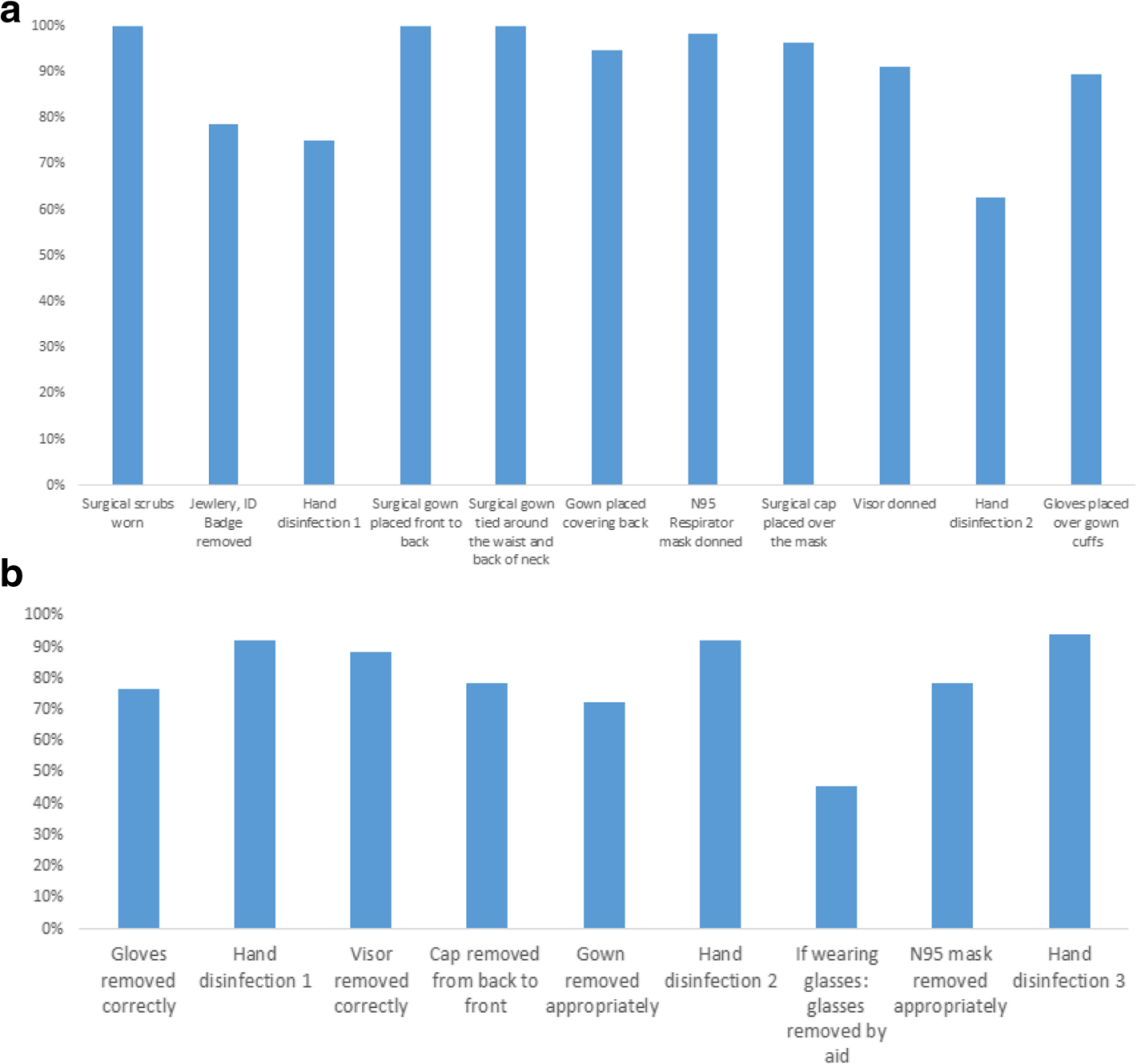
**a**. Percent of donning steps properly done by step. **b**. Percent of doffing steps properly done by step

Additional file 1 presents the PPE steps for donning. 50% donned their PPE without protocol deviations (16% entering the ED, 57% HCWs entering the ICU and 70% entering the internal medicine ward). Major omissions included failure to remove ID badges, jewelry and cell phones; failure to disinfect hands in at least one of the required steps, and neglecting to don gloves over cuffs**.** Figure 1A refers to donning by step. During donning, 41% HCWs failed to use hand sanitizer in at least one step. 32% of HCWs in the ED, failed to don gloves properly.

**Fig. 2:**
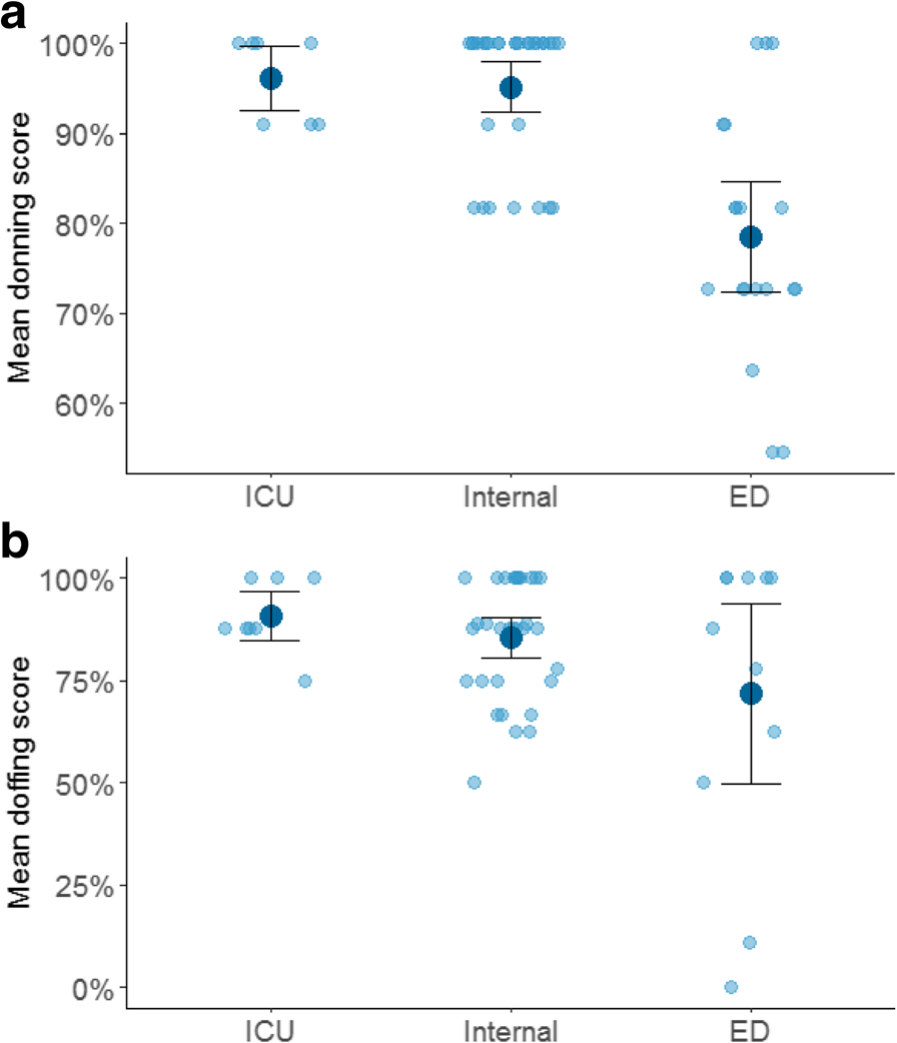
**a**. Mean donning score by department. Individual points (light blue) indicate individual scores; large points (dark blue) indicate mean score; error bars indicate confidence intervals; **b**. Mean doffing score by department. Individual points (light blue) indicate individual scores; large points (dark blue) indicate mean score; error bars indicate confidence intervals

We used a Generalized Linear Model (GLM) with quasibinomial distribution and a logit link function to predict donning mean score based on department type and worker type. We found that the emergency department had a significantly lower mean donning score (ED: 78%, Internal: 95% ICU: 96%, β = − 1.2, SE = 0.29, *p* < 0.001). Figure 2A shows the donning score by department. No differences were found between worker types.

Overall, 37% doffed their PPEs appropriately. Figure 1B refers to doffing by step. Gaps were observed in all steps; HCWs neglected to remove gloves, visor, cap, gown and N95 mask properly and failed to disinfect hands in at least one of the 3 steps. While doffing, 12% of all HCWs observed failed to disinfect their hands in at least one of 3 of the steps (steps 2, 6 and 8). Most cases of failure to disinfect hands in one of the 3 steps took place in the ED (73%). Of the 11 HCWs wearing glasses, 5 (45%) appropriately doffed by having someone else remove the glasses before removing the N-95 mask. 78% of all observed HCWs removed the N95 respirator mask appropriately.

We used a Generalized Linear Model (GLM) with quasibinomial distribution and a logit link function to predict donning mean score based on department type and worker type. The emergency department had a significantly lower mean doffing score (ED: 72%, Internal: 85% ICU: 91%, β = − 0.82, SE = 0.35, *p* = 0.02). Figure 2B shows the doffing score in the different departments. No differences were found between the worker types.

## Discussion

In our observational study of donning and doffing steps in COVID-19 departments, we report the overall rate for fully correct donning and doffing to be 44%.

This concerning finding is similar to prior studies observing PPE use (5, 11, 16, 17). Kwon at al reported that protocol deviations were common in both donning and doffing, and found that 100% of Ebola Virus Disease HCWs committed at least one PPE protocol deviation during doffing and 27% while donning (5). Casalino et al. reported that PPE doffing protocol deviations occurred even after a three-phase training program (11).

During the SARS-CoV-2 pandemic, and specifically within the time period in which our study took place, strategies to improve adherence had not yet been implemented. Failure to improve the success rate may have a profound effect on HCWs morbidity. Protocol deviations in donning and doffing may have multifactorial causes. Gaps between the development of Infection Protection and Control guidelines, their introduction to the target audience, and their implementation (4, 18), likely occur for a number of reasons. Firstly, HCWs may lack sufficient practice and simulation time (11, 18); a review by Houghton et al. (2020) have suggested support from managers, workplace culture, physical space, access to and trust in PPE, and a desire to deliver good patient care as additional factors to affect guidelines following (18). Secondly, high consequence infectious diseases have been shown to produce anxiety among HCWs which may hinder performance (19). Moreover, it is both intuitive and evidence based that performing medical tasks that require psychomotor skill would be more difficult when wearing PPE (1). Previous studies have assessed the impact of PPE on resuscitative efforts. Chen et al. found that wearing PPE in a resuscitation scenario significantly deteriorates the quality of chest compression and may thus deteriorate outcome and survival (20). Castle et al. concluded in their study that intubation and intravenous cannulation attempts are adversely affected by wearing PPE (21).

We found that the ED had significantly lower mean donning and doffing scores**,** potentially attributable to the suspected status of the patients, as opposed to the designated wards with COVID-19 confirmed positive patients. Moreover, fatigue from frequent PPE changes, high volume of patients with variable degrees of acuity and inappropriate staff-to-patient ratio combined with the negative impact of PPE on resuscitative efforts, as discussed before, may have a great influence on ED HCWs and explain our findings.

Our study highlights PPE donning and doffing errors. We believe there is a need for intensive practice and regular observations for appropriate PPE use, especially for ED teams. In the past, simulation and training methods were studied and significantly improved the trainee’s proficiency (11). For example, Abualenain et al. reported in their PPE simulation-based training, that the average score using checklist upon encounter suspected case of Ebola improved significantly after simulation training (22). Although beyond the scope of this current paper, yet of higher importance, is the work that has and should be done in order to better the adherence of HCWs to PPE protocols.

### Limitations

This study had several limitations. Although we assume that pre-observation performance rates were much lower, compared to the post observation rates, we did not compare pre observation and post observation rates. Moreover, our hospital used only one style of long gown PPE and protocol (see additional file 1) during the time our study took place. Other types of PPE or different protocols may influence the error rate. A recent review published in the Cochrane database summarizes the differences between overalls, long gowns and aprons and their overall donning and doffing quality (23). Varbeek et al. found that although covering more of the body was shown to protect the HCW during care for affected patients, these PPEs were associated with less comfort, and increased difficulty in donning and doffing offering more opportunities for transmission.

## Conclusions

PPE is a vital element used not only to combat pandemics but also in the daily dealing with common pathogens requiring contact precaution. Resources need to be extensively invested to assure implementation of PPE donning and doffing protocols in order to improve HCWs and patients’ protection. PPE type needs to be selected based on ease of care, comfort and training capabilities. Finally, a special focus needs to be invested in ED teams due to the special circumstances they confront with: high volume of patients with variable levels of acuity and unknown COVID-19 status.

### Authors’ contributions

The author(s) read and approved the final manuscript.

## Supplementary Information

**Additional file 1.**
