# Supplementary material for: Emergency department impaired adherence to personal protective equipment donning and doffing protocols during the COVID-19 pandemic
Source: Isr J Health Policy Res. 2021 Jul 19;10:41. doi: 10.1186/s13584-021-00477-7 (PMC8287287; doi:10.1186/s13584-021-00477-7)
Supplement: Supplementary file 1 — Additional file 1. [file 13584_2021_477_MOESM1_ESM.zip › Doffing poter.pdf]

Personal Protective Equipment (PPE) doffing steps for airborne pathogens

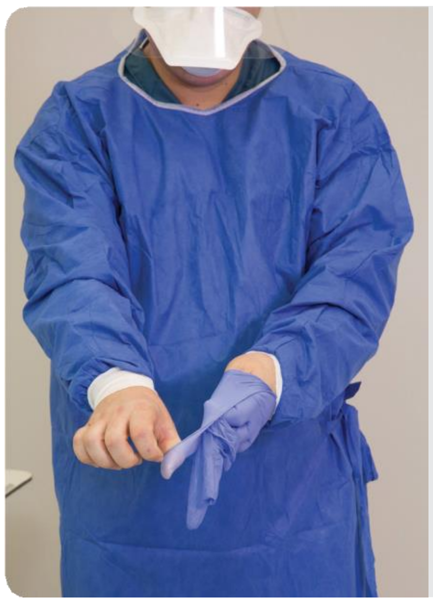

1 Remove the gloves one inside the other without touching the outside surface

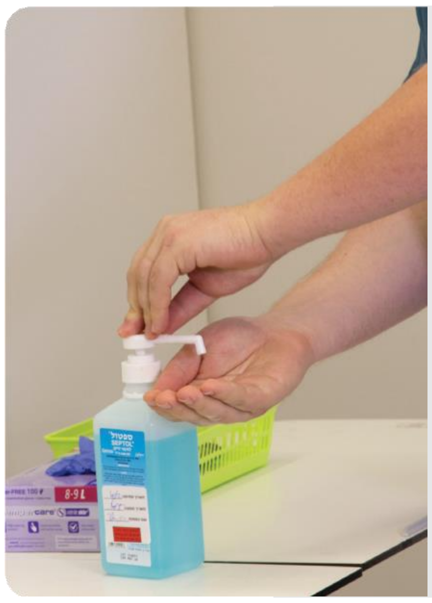

2 Disinfect your hands

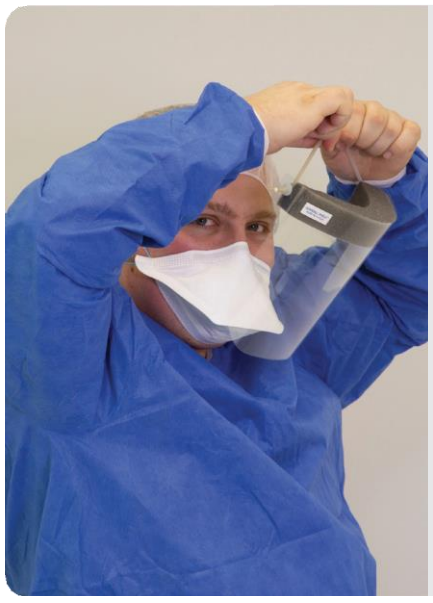

3 Remove the visor without touching the front/exposed surface of the visor and dispose into medical waste bin

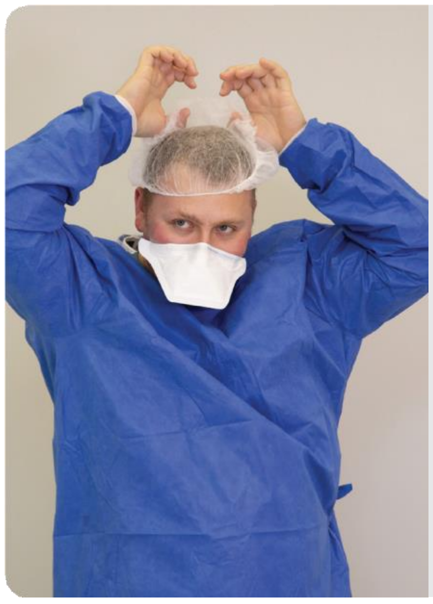

4 Remove the cap from back to front and dispose into medical waste bin

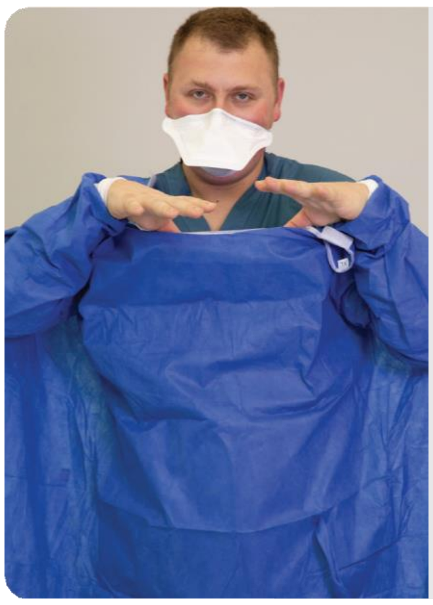

5 Remove the gown by grasping the inside surface of the collar and roll it away from you without touching the exposed surface of the gown

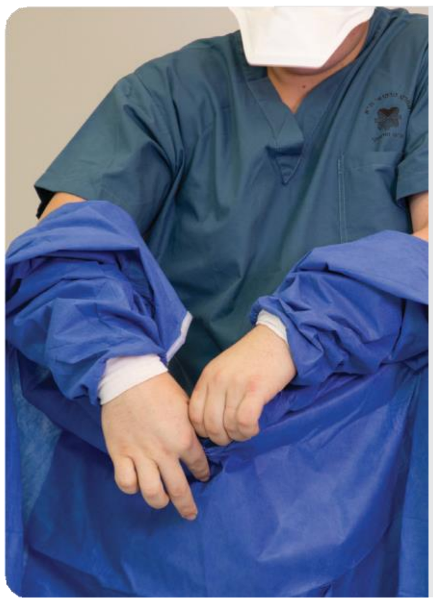

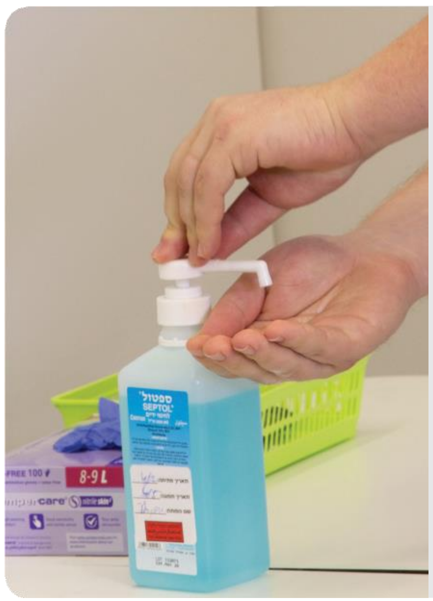

6* Disinfect your hands

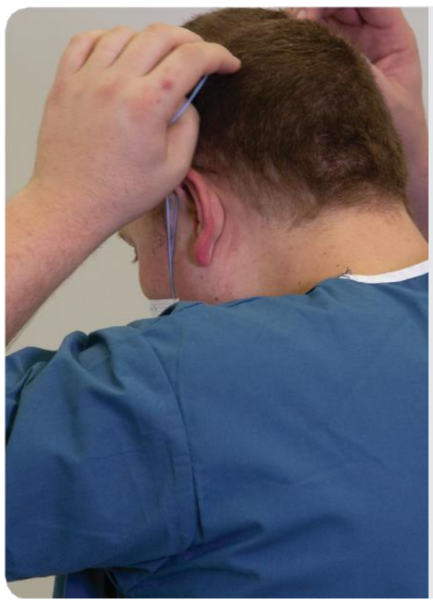

7 Remove N95 respirator mask without touching the exposed surface of the mask

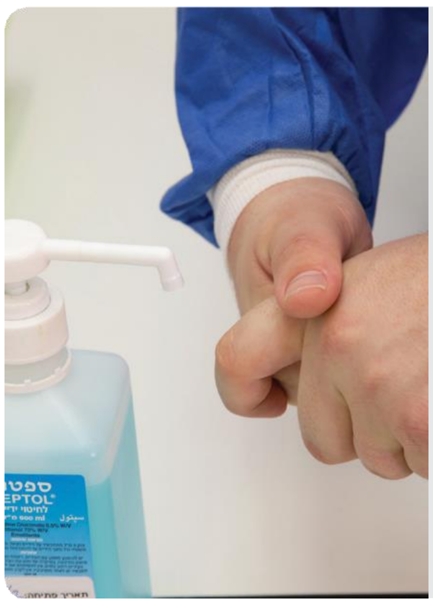

8 Disinfect your hands

***6a If wearing glasses: just before removing the N95 mask a colleague should remove your glasses**
