# Supplementary material for: Emergency department impaired adherence to personal protective equipment donning and doffing protocols during the COVID-19 pandemic
Source: Isr J Health Policy Res. 2021 Jul 19;10:41. doi: 10.1186/s13584-021-00477-7 (PMC8287287; doi:10.1186/s13584-021-00477-7)
Supplement: Supplementary file 1 — Additional file 1. [file 13584_2021_477_MOESM1_ESM.zip › Donning poster.pdf]

Personal Protective Equipment (PPE) donning steps for airborne pathogens

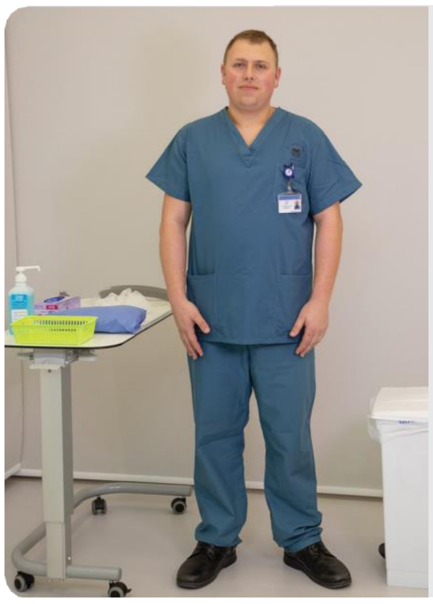

Wear surgical scrubs

1

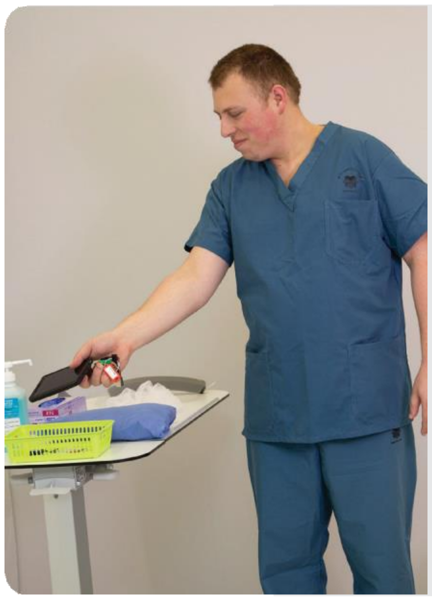

Take off jewelry and identity card, leave cell phone outside, tie up hair

2

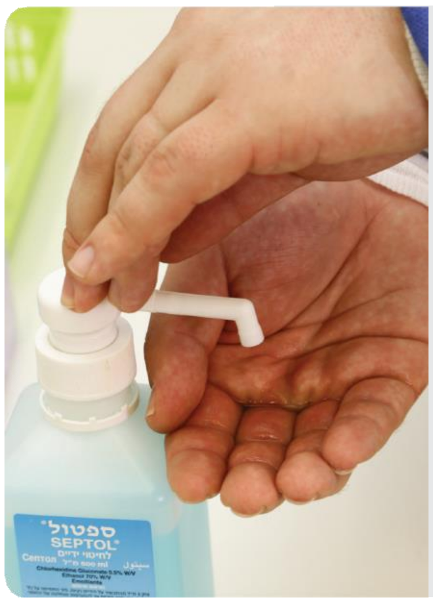

Disinfect your hands

3

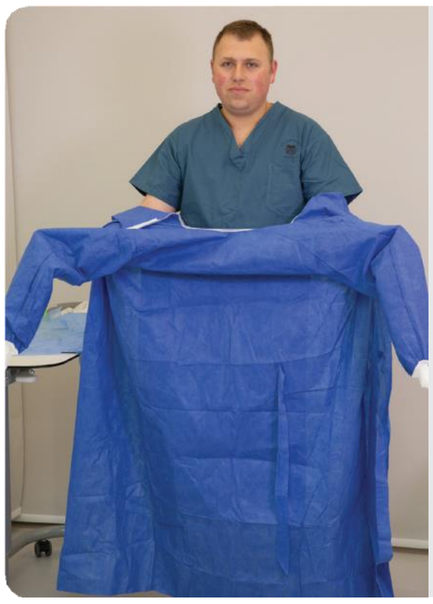

Place the surgical gown from opening to back

4

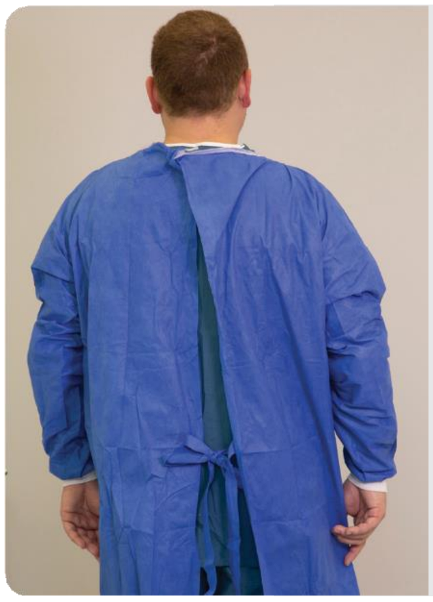

Tie the gown around the waist and in the back of the neck

5

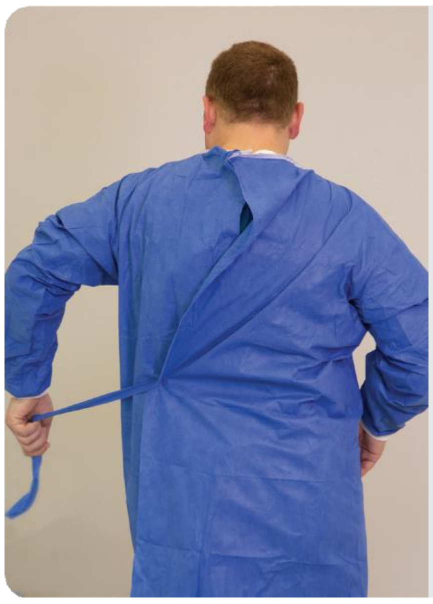

Make sure to have full back coverage

6

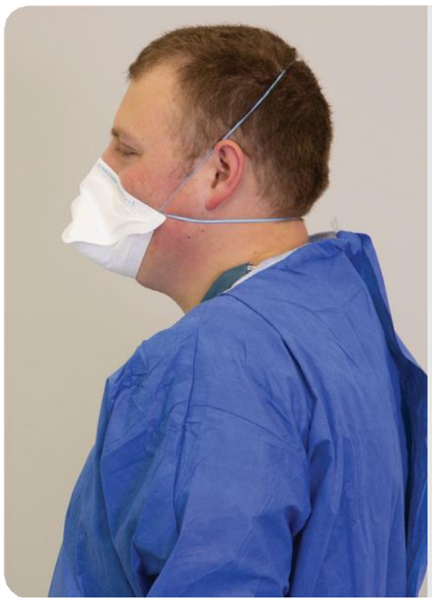

Don the N95 respirator mask and inhale twice to reassure it fits well to your face

7

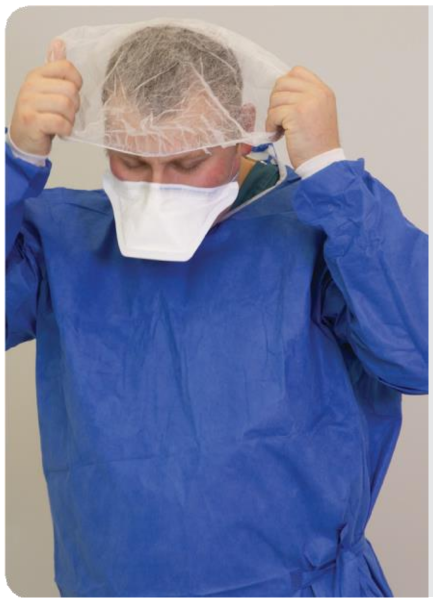

Place the surgical cap over the mask so it will cover your hair

8

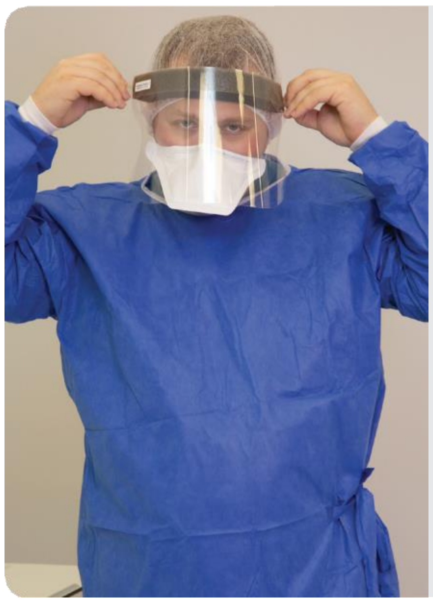

Put on the visor

9

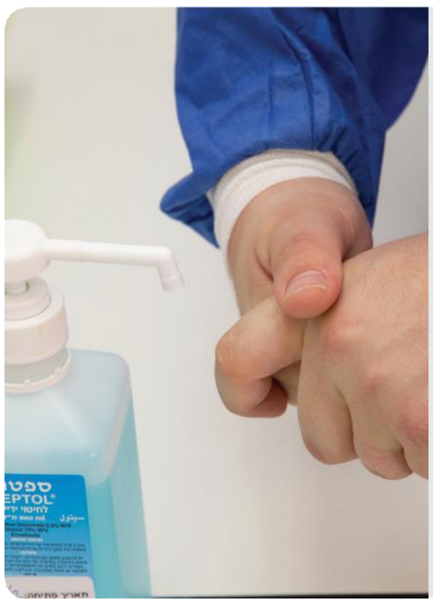

Disinfect your hands

10

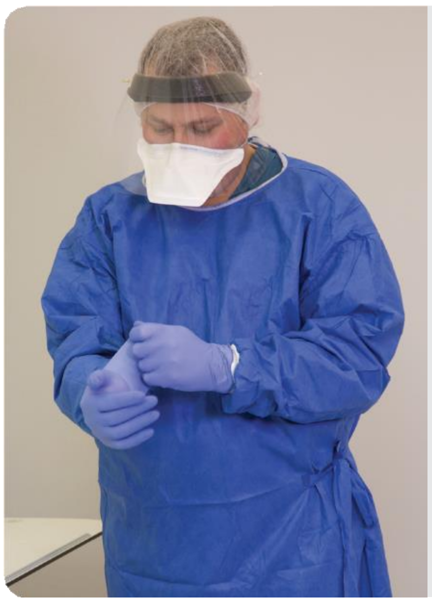

Put on the gloves above the white part of the gown's sleeves

11
